# P-801. Epidemiology and Clinical features of 101 Patients with *Aeromonas* Bacteremia

**DOI:** 10.1093/ofid/ofae631.993

**Published:** 2025-01-29

**Authors:** Daisuke Masuda, Keiichi Furukawa

**Affiliations:** Asahi General Hospital, Asahi city, Chiba, Japan; Asahi General Hospital, Asahi city, Chiba, Japan

## Abstract

**Background:**

Recently pts with *Aeromonas* bacteremia have been increasingly recognized at our hospital. We investigated epidemiology and clinical features of pts with *Aeromonas* bacteremia at Asahi General Hospital (980 beds, acute care general hosp. Japan).

**Methods:**

We retrospectively reviewed the medical records of all pts with positive blood cultures for *Aeromona*s from Jan1, 2018 to Sept30, 2023. We investigated characteristics of the pts , history of well water use , focus of infections, species , antimicrobial susceptibility, therapy, and 30-day mortality.

**Results:**

Total 101 pts with *Aeromonas* bacteremia were included. We had average 20 *A*. bacteremia cases per a year and the number was much higher than other reports. Well water was used at home in 66%(21/32) of the pts. Male 69%(p=0.23),Median age 79 (42-97), Almost all pts(98%) had baseline diseases : malignancy 54%, cancer of biliary tract or liver 36%, diabetes mellitus 26%,steroid or immunosuppressant use 5%chr. renal diseases 5%, chr. liver disease 3%. Community acquired infect. was 81%. Hepatobiliary tract infect. was the most common 77%, (cholangitis 67%, cholecystitis7%, liver abscess 2%), septic shock unknown focus 6%, urinary infect. 3%, peritonitis 2%, skin soft tissue infect. 2% , others 11%. *A.caviae* was predominant 63%, *A.veronii* 20%, *A.hydrophila*17%. Mixed bacterial infect. 58%. Antimicro. suscept.(%) : PIPC/TAZ97, CMZ78, CTX89, CFPM100, MEPM97, LVFX100. The 30-days mortality rate(%) was 20 (21/101) in total and was relatively high in PIPC/TAZ therapy 34(15/44), followed by CTX 17, CTRX17, MEPM17, CMZ8,and CFPM0, LVFX0, CPFX0. The 30-days mortality rates by species(%): *A.hydrophila* 29, *A.veronii*25, *A.caviae* 17, Mixed infect. 22.

**Conclusion:**

We had many pts with *Aeromonas* bacteremia , which might be related to well water use of many pts in our district. Most of the pts were elderly, having baseline diseases including cancer(54%) and hepatobiliary tract infect. was most common. The 30-day mortality rate was lowest in the therapy group with CFPM or LVFX .

**Disclosures:**

**All Authors**: No reported disclosures

